# Application of deep learning in crop research: From genomics to phenomics

**DOI:** 10.1002/tpg2.70268

**Published:** 2026-06-22

**Authors:** Zefeng Wu, Yali Sun, Qian Luo, Jiaping Wei, Junmei Cui, Yan Fang, Yining Niu, Zhaohong Li, Xiaolin Wang, Zigang Liu

**Affiliations:** ^1^ State Key Laboratory of Aridland Crop Science Gansu Agricultural University Lanzhou China; ^2^ Key Laboratory of Animal Genetics, Breeding and Reproduction of Shaanxi Province, College of Animal Science and Technology Northwest A&F University Yangling China; ^3^ College of Agriculture South China Agricultural University Guangzhou China

## Abstract

Deep learning, as a pivotal branch of machine learning, has demonstrated remarkable potential in advancing crop science by effectively integrating genomics and phenomics. This review systematically outlines the application of diverse deep learning architectures—such as convolutional neural networks, recurrent neural networks, and transformers—across key crop genomic tasks, including gene expression prediction, alternative splicing analysis, *cis*‐regulatory element identification, epigenomic profiling, and genome‐based trait prediction. In phenomics, these models facilitate high‐throughput extraction of crop phenotypic traits from multispectral, unmanned aerial vehicle, and ground‐based imagery, supporting yield forecasting, disease diagnosis, and stress response monitoring. We critically evaluate the performance and limitations of each model type across tasks, considering trade‐offs between complexity, accuracy, and interpretability, to offer practical guidance for crop researchers. Additionally, the review addresses major challenges in deploying deep learning—such as data scarcity, model transparency, and computational demands—and proposes future pathways to enhance model generalizability, multimodal data integration, and applications in intelligent breeding and sustainable agriculture.

## Plain Language Summary

Deep learning, a powerful form of artificial intelligence, is opening new doors in crop science by connecting the dots between a plant's genetic code and how it actually grows in the field. In this review, we explore how different deep learning models help scientists tackle two big challenges: predicting gene behavior and extracting useful information from crop images captured by drones and cameras. These models can forecast traits like yield and disease resistance based on genetic data, while also enabling early detection of pests, diseases, and stress responses from field images. We also compare the strengths and weaknesses of various approaches to help researchers choose the right tool for their needs. Although challenges like data limitations and high computing demands remain, deep learning holds great promise for making crop breeding smarter and agriculture more sustainable.

**Abbreviations**
ATAC‐Seq: assay for transposase‐accessible chromatin with sequencing
BLUP: best‐linear unbiased prediction
CNN: convolutional neural network
CRISPR: clustered regularly interspaced short palindromic repeats
DL: deep learning
FAIR: findable, accessible, interoperable and reusable
GAN: generative adversarial network
GBLUP: genomic best‐linear unbiased prediction
GWAS: genome‐wide association studies
HTP: high‐throughput phenotyping
LIME: Local Interpretable Model‐agnostic Explanations
LLM: large language model
LSTM: long short‐term memory network
MIAPPE: minimum information about plant phenotyping experiments
NLP: natural language processing
PDI: proximal‐distal interactions
PPI: promoter‐proximal interactions
QTL: quantitative trait locus
ResNet: residual convolutional network
RNN: recurrent neural network
SHAP: Shapley additive explanations
SNP: single nucleotide polymorphism
SR: serine/arginine‐rich
SVM: support vector machine
TFBS: transcription factor binding sites
UAV: unmanned aerial vehicle

## 1 INTRODUCTION

Plant genomes are the fundamental repository of genetic information, dictating the blueprint for growth, development, environmental adaptation, and ultimately, the yield and resilience of crops. The advent of high‐throughput sequencing technologies has catalyzed a genomic revolution, enabling the sequencing and assembly of numerous crop genomes to chromosome‐level and even complete telomere‐to‐telomere completeness (Shirasawa et al., 2021). This has shifted the paradigm from obtaining a single reference genome to constructing pan‐genomes that capture the vast genetic diversity within species. Concurrently, the field of crop phenomics has emerged to address the “phenotyping bottleneck,” leveraging advanced sensors, robotics, and imaging platforms (e.g., unmanned aerial vehicles [UAVs] and automated greenhouses) to acquire high‐dimensional phenotypic data at unprecedented scale and resolution (Furbank & Tester, 2011). These parallel advancements have generated massive, multimodal datasets, positioning crop science firmly in the era of big data, thereby laying a crucial foundation for precision breeding in crops.

The central challenge and grand goal in modern plant biology is to decipher the intricate regulatory code that links genotypes to phenotypes (Huo et al., 2024). This involves understanding how DNA sequences, through complex mechanisms involving gene expression, alternative splicing, *cis*‐regulatory elements, and epigenomic modifications (e.g., DNA methylation and histone marks), give rise to observable traits such as biomass, yield, and stress tolerance. Traditional bioinformatics tools and classical machine learning methods (e.g., support vector machine [SVM] and random forests) have provided foundational insights but are often limited in their capacity to model the nonlinear, high‐dimensional, and hierarchical relationships inherent in biological systems. Their reliance on manual feature engineering can be a bottleneck, potentially missing subtle yet biologically critical patterns encoded in raw genomic and image data.

In this context, deep learning (DL), a subfield of artificial intelligence characterized by multilayered neural networks, has emerged as a transformative force. DL offers a paradigm shift by automatically learning hierarchical feature representations directly from raw data, be it nucleotide sequences, epigenomic signals, or high‐resolution plant imagery (Tong & Nikoloski, 2021). While several insightful reviews have explored the role of DL in specific domains of crop science, such as genomic selection or high‐throughput phenotyping (HTP), they often concentrate either on the molecular level (e.g., genomics and transcriptomics) or the phenotypic level and seldom integrate the two (X. Hu et al., 2023; Murphy et al., 2024; Singh et al., 2018; H. Wang, Cimen, et al., 2020). This fragmented perspective overlooks the powerful synergies that arise when connecting genotype to phenotype. Our review seeks to bridge this gap by providing a comprehensive overview that traverses the entire spectrum, from genomics to phenomics, thereby offering a unified perspective on how DL is poised to revolutionize crop breeding and research.

In this review, we begin by elucidating the core principles of these foundational DL architectures. Subsequently, we delve into their specific applications, structuring our discussion into two major pillars: (1) Applications in plant genomics: We explore how DL is revolutionizing the prediction of gene expression levels (e.g., Z. Wang, Peng, et al., 2024; Washburn et al., 2019), deciphering the complex code of alternative splicing (e.g., Chao et al., 2024; Jaganathan et al., 2019), accurately identifying *cis*‐regulatory elements like transcription factor binding sites (TFBS) (e.g., L. Liu, Zhang, et al., 2021; W. Yan et al., 2022), and forecasting epigenomic modifications such as DNA methylation and histone marks (e.g., T. Zhao et al., 2024; X. Zhou et al., 2024). We also cover its growing role in whole‐genome prediction for accelerating crop genomic selection (e.g., K. Wang et al., 2023). (2) Applications in plant phenomics: We survey the deployment of DL for extracting meaningful information from HTP platforms. This includes its pivotal role in crop yield prediction from UAV and satellite imagery (e.g., J. Du et al., 2025; Tanaka et al., 2023), automated disease detection and diagnosis (e.g., Dong et al., 2024; Toda & Okura, 2019), and the precise quantification of a wide array of morphological and physiological traits (S. Liu, Jin, et al., 2021). Crucially, we highlight emerging studies that associate DL‐derived phenomic features with genetics to power gene discovery and breeding (e.g., J. Chen et al., 2024; Gan et al., 2025).

Throughout this review, we critically evaluate the pros and cons of different DL approaches in specific biological contexts. We dedicate significant discussion to the pressing challenges facing the field, including the substantial data requirements and quality issues, the critical need for model interpretability to gain biological insights, and the significant computational resources required. Finally, we offer a perspective on future directions, discussing the potential of foundational models, multimodal data integration, and the development of more efficient and biologically informed algorithms. Our aim is to provide a clear and critical roadmap of how DL is reshaping our ability to decode the genetic and phenotypic complexity of crops, thereby paving the way for smarter, faster, and more precise crop improvement strategies.

## 2 DL METHODS COMMONLY USED IN AGRICULTURAL APPLICATIONS

**FIGURE 1:**
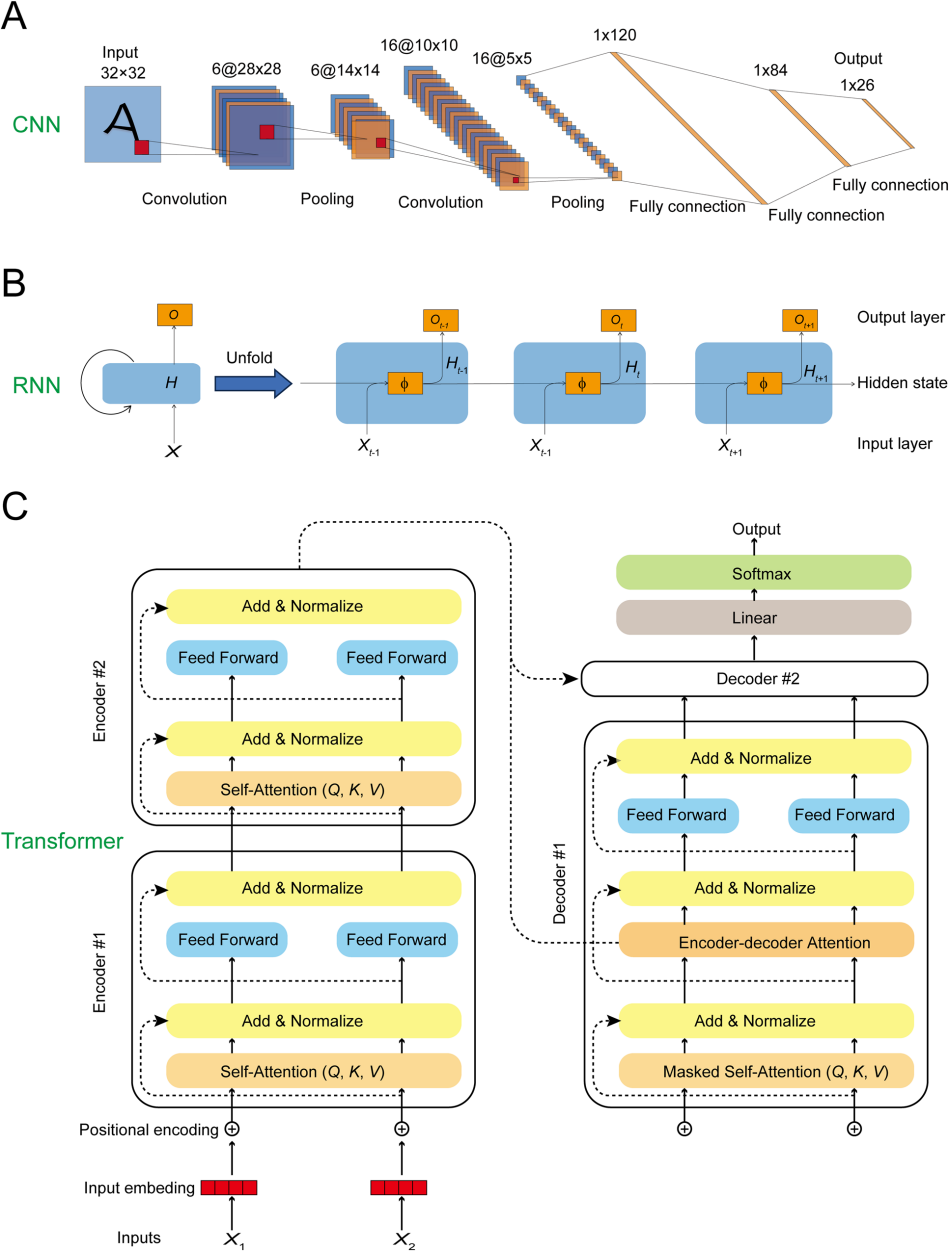
Three typical deep learning model architectures: convolutional neural network (CNN) (A), recurrent neural network (RNN) (B), and transformer (C). CNN generally consists of convolutional layers, pooling layers, and fully‐connected layers. Convolutional layers apply filters (kernels) that slide across the image, detecting local patterns (edges, textures, and shapes) via weighted sums. Each filter produces a feature map. Pooling layers (e.g., max pooling) reduce feature map dimensions, decreasing computation, controlling overfitting, and making features more translation‐invariant by summarizing local regions (e.g., taking max value). Fully connected (FC) layers interpret extracted features for classification and the final FC layer outputs scores (logits) for each possible class. For example, by constructing a CNN model using images of the 26 letters, the training process enables the model to effectively learn the distinctive features of each letter. When presented with an unseen sample of the letter A, the model can predict the probability distribution over all letter classes. If it assigns a high probability to the correct class “A,” it indicates a relatively accurate and reliable model. (B) RNN is a class of neural networks specifically designed for processing sequential data, such as text, time series, and speech signals. Unlike feed‐forward networks, RNNs process inputs in a step‐by‐step manner, where each step receives both the current input and a hidden state representing a compressed “memory” of all previous inputs. This recurrent mechanism, where the output of a step is fed back into the network, allows RNNs to model temporal dependencies and dynamic patterns over time. The core computation at each time step involves updating the hidden state, typically through a function combining the current input and the previous hidden state. This enables the network to learn context and relationships across sequences. (C) Transformer model typically consists of two main components: an encoder that processes the input sequence to extract and compress its features into a hidden representation, and a decoder that utilizes this representation to auto‐regressively generate the output sequence. Both the encoder and decoder are built by stacking multiple identical layers containing multi‐head attention and feed‐forward networks.

With the increasing development and updating of algorithms, DL has been widely used and acknowledged in different areas of research. As so far, various deep DL methods or frameworks have been applied in plants and agriculture research, and some typical models included convolutional neural networks (CNNs), recurrent neural network (RNN), long short‐term memory networks (LSTMs), generative adversarial networks (GANs), transformer, and their variations. CNNs generally consist of convolutional layers, pooling layers, and fully connected layers (Krizhevsky et al., 2017) (Figure 1A), with ability to capture local spatial hierarchies and are instrumental in identifying motifs in DNA sequences and analyzing image‐based phenotypes (Y. Jiang & Li, 2020; Shen et al., 2021). Residual convolutional network (ResNet) is an extension of CNN and it introduces residual blocks with shortcut connections (He et al., 2016). These connections allow the network to skip one or more layers, enabling the direct flow of information and gradients. This approach not only facilitates the training process but also leads to improved accuracy as the model depth increases. Currently, the concept of residual connections is extensively applied in deep neural networks to address the vanishing or exploding gradient problem (G. Xu et al., 2025). A RNN is a class of neural network architectures designed to handle sequential data (Lipton et al., 2015) (Figure 1B). Sequential data are data where the order of the elements really matters, such as time‐series data (e.g., stock prices over time), natural language sentences (words in a specific order), and audio signals. RNNs have the ability to process such data by maintaining an internal state that captures information about the sequence seen so far. LSTMs are a specialized type of RNN. They were developed to address the limitations of traditional RNNs, particularly the problem of vanishing and exploding gradients when dealing with long‐sequence data. LSTMs have become a cornerstone in many sequential data‐processing tasks such as natural language processing (NLP), speech recognition, and time‐series analysis. RNNs and LSTMs are tailored for sequential data, modeling temporal phenomic patterns or dependencies in nucleotide sequences (Lipton et al., 2015). The transformer model, proposed in the 2017 paper “Attention is all you need” (Vaswani et al., 2017), is a revolutionary neural network architecture in the field of NLP and has been widely applied in various other fields (Figure 1C). The transformer architecture, with its self‐attention mechanism, excels at capturing long‐range dependencies in both genomes and complex scenes, revolutionizing tasks from gene expression prediction to fine‐grained phenotyping (Consens et al., 2025; Vaswani et al., 2017). Furthermore, GANs are being explored to generate synthetic data and augment limited training sets (Goodfellow et al., 2014), while graph neural networks (GNNs) are increasingly applied to model relational and spatial structures in agricultural systems, such as crop field layouts, gene regulatory networks, or the soil–plant–atmosphere continuum (Gupta & Singh, 2023; Mahareek et al., 2025; W. Zhang & Guo, 2026). The application of these DL models is rapidly transforming agricultural research, and a growing number of studies are now combining the aforementioned model types—such as CNNs with RNNs, transformers with CNNs, or GANs with CNNs—to leverage their complementary strengths for specific agricultural tasks.

## 3 APPLICATIONS OF DL IN PLANT GENOMICS

### 3.1 Gene expression prediction

**FIGURE 2:**
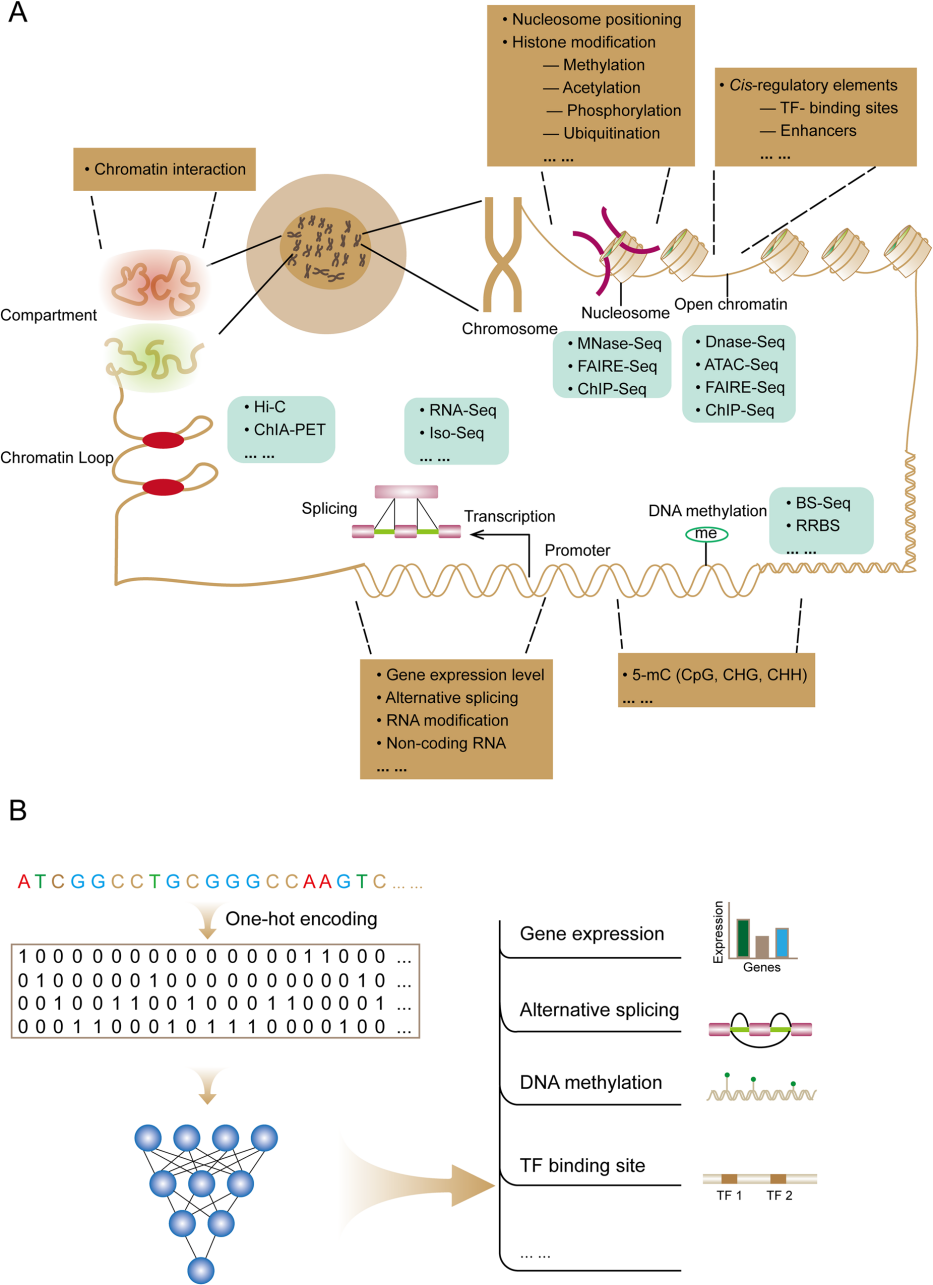
Principle of gene regulation in plants. (A) Gene expression regulation in plants. Regulation of gene expression can occur at multiple levels, such as nucleosome positioning, histone modifications, dynamic binding of transcription factors, DNA methylation, alternative splicing, and the spatial architecture of chromatin. Brown boxes indicate different types of gene regulation, while blue boxes show the corresponding sequencing or profiling techniques. (B) Typical deep learning models take DNA sequences as input, utilizing one‐hot encoding for training to predict outputs such as gene expression levels, splicing patterns, and epigenomic features.

Understanding plant gene expression regulation has long been a priority for plant scientists. Newly developed methods—frequently based on next‐generation sequencing and advanced computational approaches–are now illuminating the gene regulatory logic employed by plants (Michael & VanBuren, 2015). Gene expression is the process by which the information encoded in a gene is turned into a function. This mostly occurs via the transcription of RNA molecules that code for proteins or noncoding RNA molecules that serve other functions. Gene expression is regulated by multiple factors, including DNA methylation, histone modifications, chromatin accessibility, and spatial chromatin interactions (Cramer, 2019; H. Zhang & Zhu, 2025; Y. Zhang et al., 2024) (Figure 2). Gene expression prediction is a crucial task in understanding biological processes, disease mechanisms, and developing personalized medicine. DL techniques have emerged as powerful tools in this field, offering the potential to handle complex genomic data and extract meaningful patterns regarding to gene expression prediction. Similar to image recognition, various DL models are gradually being applied to the task of predicting gene expression based on DNA sequences. In the genomes of humans and animals, some relevant studies have been reported (Barbadilla‐Martinez et al., 2025; Y. Chen et al., 2016; J. Zhou et al., 2018), but these studies are not the focus of this review.

**FIGURE 3:**
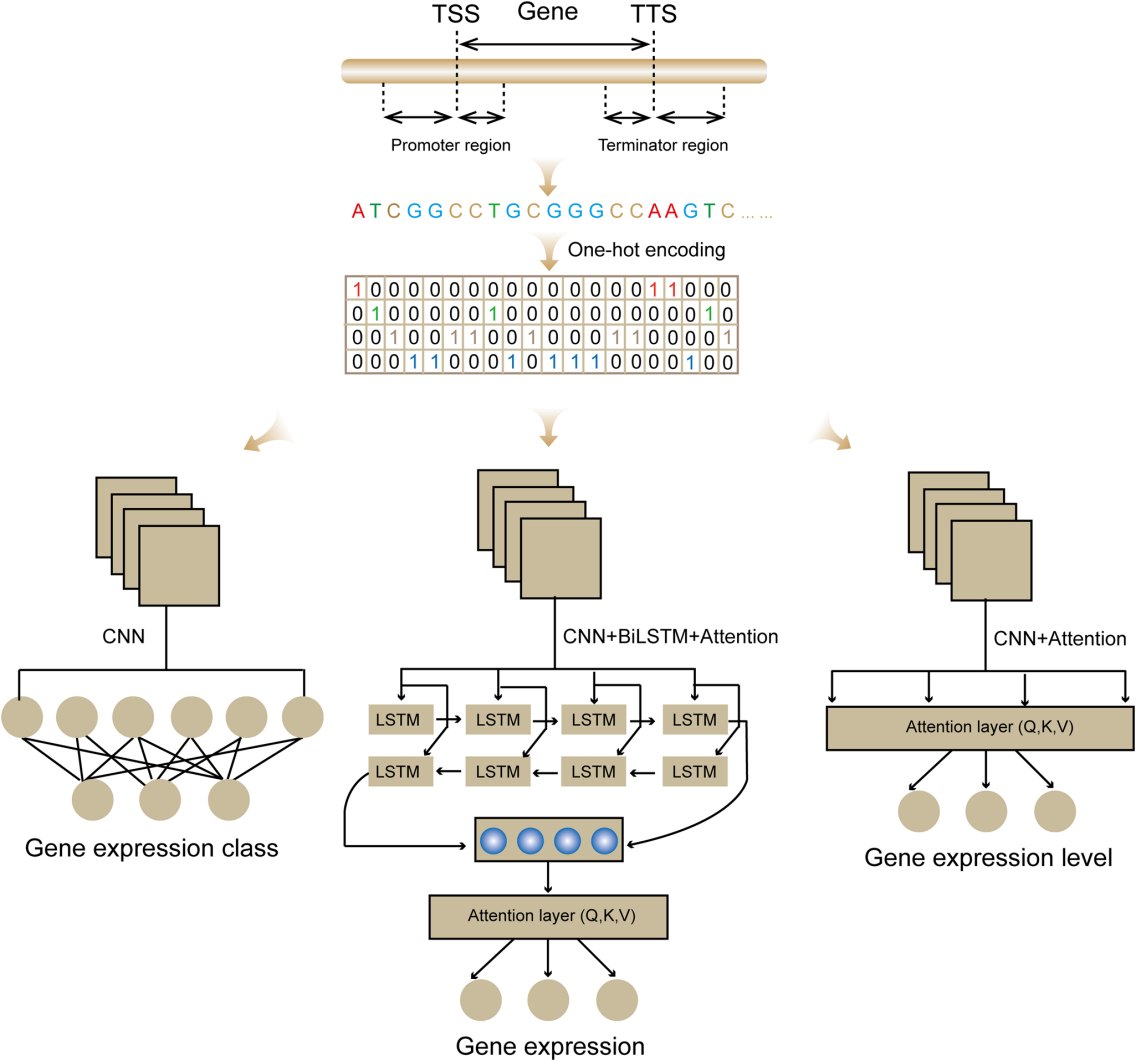
Schematic of deep learning (DL)‐based gene expression prediction in crops. This schematic illustrates a general pipeline for predicting gene expression levels. DNA sequences of predetermined lengths are first extracted from genomic regions such as promoters and/or terminators. These sequences are subsequently transformed into a one‐hot encoded matrix to serve as input features. Various DL architectures then process this matrix to perform the classification or regression task of modeling gene expression. A common architectural pattern among these deep learning models for crop gene expression prediction is the use of convolutional neural networks (CNNs), either in isolation or as the foundational component in a hybrid architecture, which is often followed by bidirectional long short‐term memory network (BiLSTM) or attention layers for further sequence analysis. TSS, transcription start site; TTS, transcription terminate site.

In plants, several studies have been conducted to model gene expression level based on DNA sequence using DL methods (Figure 3; Table 1). Washburn et al. (2019) first train CNN models with DNA sequences surrounding annotated transcription start and end site of genes to predict gene expression level in maize (*Zea mays* L.). More recently, Peleke et al. (2024) trained interpretable CNN models on gene flanking regions of *Arabidopsis* (*Arabidopsis thaliana* (L.) Heynh.), tomato (*Solanum lycopersicum* L.), sorghum (*Sorghum bicolor* (L.) Moench), and maize to predict maximum gene expression levels in leaves and roots with over 80% accuracy. They also employed the DeepLIFT algorithm to interpret the model's predictions and extracted expression prediction motifs using the TFMoDISco algorithm. It could identify the conserved and specific regulatory sequence features across different species and effectively predict the impact of genetic variations on gene expression. In addition to sequence only, other features were also integrated to DNA sequences to predict gene expression in plants. For example, Z. Wang, Peng, et al. (2024) developed a deep‐learning model named DeepCBA for predicting gene expression levels in maize based on chromatin interaction sequences. This model incorporates a CNN, a bidirectional long short‐term memory network, and a self‐attention mechanism. Compared with existing models, DeepCBA exhibits higher accuracy in both gene expression classification and the prediction of gene expression values. When using promoter‐proximal interactions (PPI), proximal‐distal interactions (PDI), and PPI + PDI interactions to predict gene expression, the average Pearson correlation coefficients are 0.818, 0.625, and 0.929 respectively. The accuracy of DeepCBA in predicting changes in gene expression values was further verified through promoter saturation mutation experiments. Another research utilized 600,000 genes from 17 plant species and 6256 sets of transcriptome data to develop a deep‐learning model named PhytoExpr (T. Li, Xu, et al., 2024). This model takes the DNA sequences of the proximal transcriptional regulatory regions (a 5‐kb promoter and a 5‐kb terminator) as input to predict the median expression level of genes and the species from which the sequences originate. When adopting the transformer architecture and multitask learning, the model achieves higher accuracy in both mRNA abundance prediction and species‐origin prediction of sequences. The model can make reliable predictions for unseen gene families in unseen species except *Chlamydomonas reinhardtii*. It can also evaluate the impact of each base in the transcriptional regulatory region of every maize gene on gene expression. Different from one step modeling based on DNA sequences, Akagi et al. (2022) adopted two steps ML modeling to predict the gene expression patterns, with addressing the importance of *cis*‐regulatory elements in gene expression regulation Specially, they first trained a model using the cistrome dataset of *Arabidopsis* to predict potential *cis*‐regulatory elements in promoter regions of tomato genes. Then, based on the TF‐binding patterns, they built second CNN model for predicting differential expression patterns in ripening tomato. Through the feature visualization technology of the CNN, they identified the key nucleotide residues and verified the predictions of the DL model using electrophoretic mobility shift assay and transient reporter gene analysis.

**TABLE 1:** A summary of deep learning models used in plants or crops study.

Tasks	Model name	Model architecture	Accuracy	Species	Source code	Computation frame	Publication
Gene expression prediction	p_strength_prediction	CNN	0.75–0.94	*Z. mays*	https://bitbucket.org/bucklerlab/p_strength_prediction/src/master/	TensorFlow (Keras)	(Washburn et al., 2019)
	CisDecoding_cistrome	CNN	0.956	*S. lycopersicum*	https://github.com/Takeshiddd/CisDecoding_cistrome	Keras	(Akagi et al., 2022)
	PhytoExpr	CNN, transformer	0.85–0.95	17 plants	https://doi.org/10.6084/m9.figshare.24417076.v1	TensorFlow (Keras)	(T. Li, Xu, et al., 2024)
	DeepCRE	CNN	>0.8	*A. thaliana*, *S. lycopersicum*, *S. bicolor*, and *Z. mays*	https://github.com/NAMlab/DeepCRE	TensorFlow (Keras)	(Peleke et al., 2024)
	DeepCBA	CNN + BiLSTM + attention	0.625–0.925	*Z. mays*	https://github.com/Jie‐Lii/DeepCBA	TensorFlow (Keras)	(Z. Wang, Peng, et al., 2024)
TFBS prediction	TSPTFBS	CNN	0.991	*A. thaliana*	https://github.com/liulifenyf/TSPTFBS	TensorFlow (Keras)	(L. Liu, Zhang, et al., 2021)
	PlantBind	CNN + BiLSTM	0.961–0.973	*A. thaliana*	https://github.com/wenkaiyan‐kevin/PlantBind	PyTorch	(W. Yan et al., 2022)
	TSPTFBS 2.0	DenseNet	0.979–0.996	*Z. mays*, *A*. *thaliana*, and *O. sativa*	https://github.com/liulifenyf/TSPTFBS‐2.0	TensorFlow (Keras)	(H. Cheng et al., 2023)
	DeepTFBS	ResNet + BiLSTM	0.964	*A. thaliana*	https://github.com/cma2015/deepTFBS	TensorFlow (Keras)	(Zhai et al., 2025)
DNA methylation	SMEP	CNN	0.95	*O. sativa*	https://github.com/BRITian/smep	TensorFlow (Keras)	(Y. Wang, Zhang, et al., 2021)
	DeepFDML	CNN + transformer	0.82	*G. hirsutum*	–	PyTorch	(T. Zhao et al., 2024)
	PlantDeepMeth	CNN, GRU	>0.8	*A. thaliana* and *B. rapa*	https://gitee.com/Bioinformaticslab/PlantDeepMeth	TensorFlow (Keras)	(Z. Guo et al., 2025)
Histone modification	Osei	CNN	∼0.904	*O. sativa*	https://github.com/compbioNJU/Osei	PyTorch	(X. Zhou et al., 2024)
Genomic prediction or selection	Yield‐Prediction‐DNN	DNN	0.78	*Z. mays*	https://github.com/saeedkhak92/Yield‐Prediction‐DNN	Tensorflow	(Khaki & Wang, 2019)
	DNNGP	CNN	0.63	*Z. mays* and *T. aestivum*	https://github.com/AIBreeding/DNNGP	TensorFlow (Keras)	(K. Wang et al., 2023)
	TrG2P	CNN	0.42–0.94	*Z. mays*, *O. sativa*, and *T. aestivum*	https://github.com/lijinlong1991/TrG2P	TensorFlow	(J. Li, Zhang, et al., 2024)
	Cropformer	CNN + attention	0.76–0.92	*Z. mays*	https://github.com/jiekesen/Cropformer	PyTorch	(H. Wang et al., 2025)
	GEFormer	gMLP + attention	0.4–0.70	*Z. mays*, *O. sativa*, and *T. aestivum*	https://github.com/Deep‐Breeding/GEFormer/tree/main/GEFormerV1.0	Pytorch	(Yao et al., 2025)
Yield estimation	rice_yield_CNN.	CNN	0.57–0.69	*O. sativa*	https://github.com/r1wtn/rice_yield_CNN	PyTorch	(Tanaka et al., 2023)
	–	DNN, LSTM, CNN	0.57–0.67	*O. sativa*	https://zenodo.org/records/12789454	MATLAB	(H. Zhou, Huang, et al., 2025)
	yield_prediction	LSTM	0.88–0.93	*T. aestivum*	https://github.com/limitlesszang/yield_prediction	TensorFlow (Keras)	(E. Cheng et al., 2022)
	RGBNet, DSMNet, and RGB‐DSMNet	CNN	0.59–0.95	*G. max*	–	PyTorch	(Okada et al., 2024)
	SoyNet/SoyNet‐Res	ResNet	0.60	*G. max*	https://gitlab.com/zlyzly28/plant‐phenomics	Paddle	(L. Zhou, Han, et al., 2025)
	–	R‐CNN	0.86	*M. domestica*	–	TensorFlow	(Apolo‐Apolo et al., 2020)
	Yolo‐v5	ResNet	0.85	*C. sinensis*	https://github.com/bubbliiiing/yolov5‐pytorch	PyTorch	(S. Wang, Yu, et al., 2024)
	VGG‐16, ResNet‐50	ResNet	0.76–0.79	*F. ananassa*	–	TensorFlow (Keras)	(C. Zheng et al., 2022)
	–	CNN + transformer	0.70	*T. aestivum*	–	PyTorch	(J. Du et al., 2025)
	SSA‐LSTM‐transformer (SLTF)	transformer	0.72	*T. aestivum*	–	TensorFlow	(F. Guo et al., 2024)
Disease detection	–	CNN	0.99	14 species	https://github.com/totti0223/lucid4keras	TensorFlow (Keras)	(Toda & Okura, 2019)
	PDDM	CNN	–	63 species	http://plantpad.samlab.cn.	–	(Dong et al., 2024)
	LWDSC‐SA	CNN	0.98	14 species	–	–	(Batool et al., 2025)
	ResNet‐CAM	CNN	0.829	*M. domestica*	–	PyTorch	(L. Zhou et al., 2023)

*Note*: “–” denotes not available.

Abbreviations: BiLSTM, bidirectional long short‐term memory network; CNN, convolutional neural network; DNNGP, deep neural network genomic prediction; gMLP, gated multilayer perceptron; GRU, gated recurrent unit; LSTM, long short‐term memory network; LWDSC‐SA, depthwise separable convolution with spatial attention; PPDM, plant disease diagnosis multimodal; ResNet, residual convolutional network; SMEP, smart model for epigenetics in plants; TFBS, transcription factor binding sites.

Despite these advances, several limitations remain, pointing to clear directions for future work. For example, most current models are designed to predict bulk or maximum gene expression levels, often overlooking the tissue‐specific and spatiotemporal dynamics essential for understanding plant development and environmental adaptation. This limitation is compounded by the fact that many models are trained on limited tissue types or conditions, restricting their generalizability. Furthermore, there is a heavy reliance on predefined regulatory regions (e.g., promoters and terminators), while the role of distal regulatory elements and 3D chromatin architecture remains underexplored in crops. To address these gaps, future efforts should prioritize the prediction of tissue‐ and cell type‐specific expression in crops. This will require integrating multi‐omics data—such as chromatin accessibility (assay for transposase‐accessible chromatin with sequencing [ATAC‐Seq]), histone modifications, and DNA methylation—in a cell‐type‐resolved manner, along with the incorporation of single‐cell transcriptomic data to capture finer regulatory states.

### 3.2 Alternative splicing prediction

Alternative splicing is a critical posttranscriptional mechanism that enables the generation of multiple mRNA isoforms from a single gene, significantly increasing proteomic and phenotypic diversity (Baralle & Giudice, 2017; Wright et al., 2022). Studies have shown that alternative splicing has a central role in the definition of plant fitness plasticity to stressful conditions, and exploring alternative splicing events is important for crop breeding resilience to abiotic stresses (Y. Hu et al., 2020). Alternative splicing dynamics in plant adaptive responses to stress was reviewed by Alhabsi et al. (2025). The basic mechanism of alternative splicing involves the differential joining of exons and introns in pre‐mRNA (McManus & Graveley, 2011). Regulatory elements, including *cis*‐acting elements (such as splicing enhancers and silencers) on the pre‐mRNA sequence and *trans*‐acting factors (such as splicing factors), work together to determine the splicing pattern. For example, serine/arginine‐rich (SR) proteins bind to splicing enhancers and promote exon inclusion, while heterogeneous nuclear ribonucleoproteins bind to splicing silencers and often lead to exon skipping. The research on the molecular mechanisms of these alternative splicing events provides important theoretical basis for predicting alternative splicing events by only providing genomic information in new species.

Before the advent of deep‐learning‐based methods, traditional computational approaches for predicting alternative splicing mainly relied on sequence‐based features and applied mainly on mammalian genome. For instance, SVMs were trained on various features such as the conservation of splice sites, the length of exons and introns, and the presence of *cis*‐acting elements to predict splicing sites in human genes (Busch & Hertel, 2015). In addition, different nucleotide encoding methods have also been explored for SVM‐based splice site prediction in human genes (Huang et al., 2006). However, these traditional methods have limitations. For example, they often require a large amount of manual feature engineering, and their ability to capture complex, nonlinear relationships in the splicing data is limited.

**FIGURE 4:**
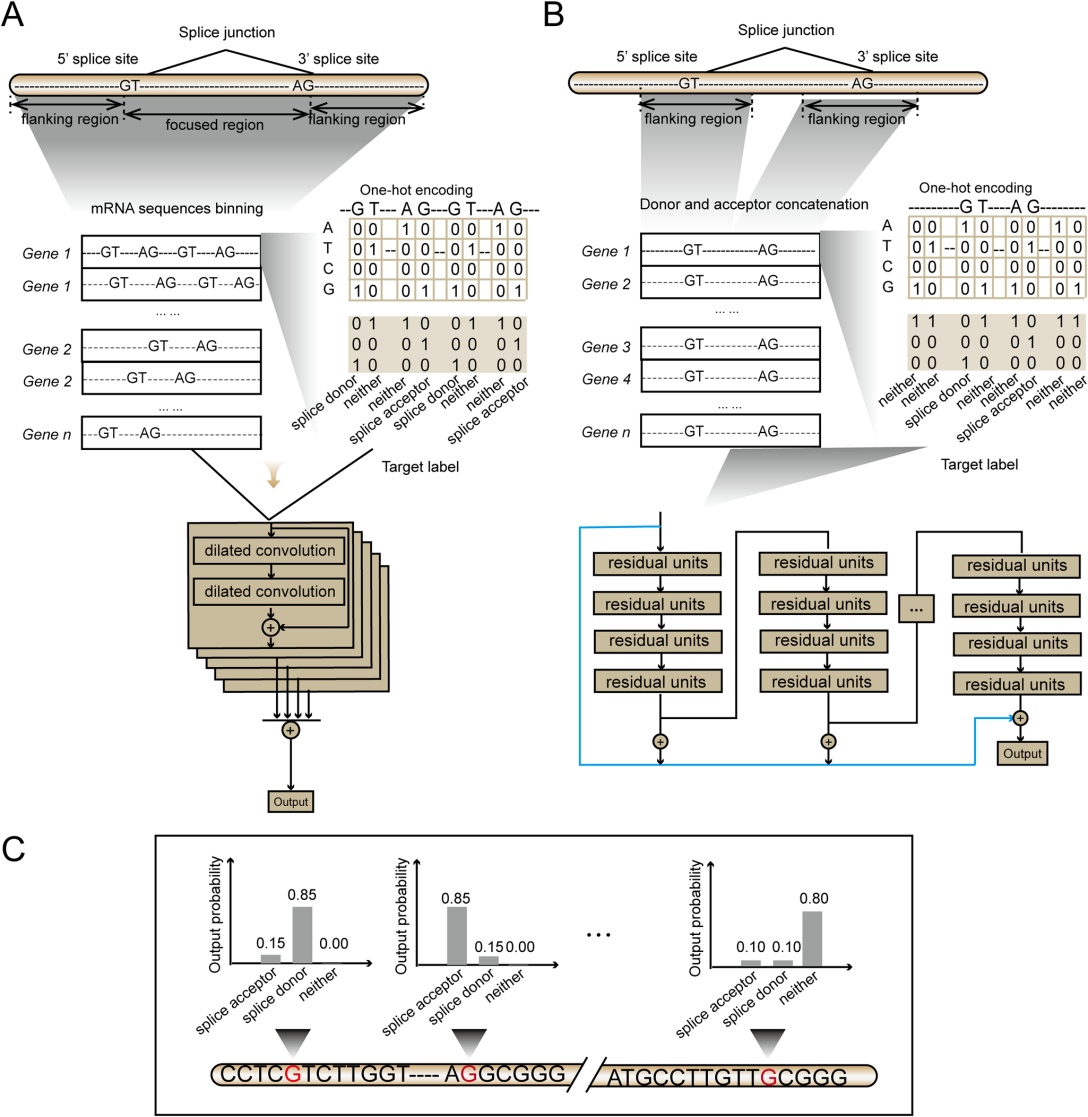
Two representative methods in gene alternative splicing code prediction based on deep learning models. (A) A schematic of SpliceAI model reported by Jaganathan et al. (2019). SpliceAI takes primary mRNA sequence as input and splitted into length‐fixed sequneces, which includes a central region or focused region flanked by extensive contextual sequences. The network architecture of spliceAI consists of 32 dilated convolutional layers that can recognize sequence determinants spanning very large genomic distances. For the central region, it predicts the probability of each nucleotide being a donor site, acceptor site, or neither. (B) A schematic of spam model reported by Chao et al. (2024). Spam uses a 400‐nt sequence centered around each end of an intron and learns the splicing pattern at the junction level. Similar with SpliceAI, it also predicts the probability of each nucleotide of the input being a donor site, acceptor site, or neither (C).

Recent advances in DL have provided powerful tools for predicting alternative splicing events and understanding their regulatory mechanisms directly using the DNA sequences surrounding splicing sites. CNNs (or deep neural networks [DNNs]) were first used to identify splicing regulatory motifs from DNA or RNA sequences in model organisms. In plants, they were initially applied to predict alternative splicing in *Arabidopsis*, followed by rice (Albaradei et al., 2020; Fernandez‐Castillo et al., 2022; Zuallaert et al., 2018). However, standard CNNs focus on local features and suffer from degradation as layer depth increases, where performance declines beyond a certain point. For this issue, the ResNets can effectively alleviate the degradation problem, which enable the network to learn richer features and better retain input information. For example, SpliceAI, a CNN‐based model consisting of 32 dilated convolutional layers that can recognize sequence determinants spanning very large genomic distances. It could predict splice junctions and alternative splicing events in a primary mRNA sequence with high accuracy (Jaganathan et al., 2019). Particularly, SpliceAI takes a long DNA sequence (e.g., 15 kb nucleotides) as input, which includes a central region (e.g., 5 kb) flanked by extensive contextual sequences. For the central region, it predicts the probability of each nucleotide being a donor site, acceptor site, or neither, enabling precise splice junction identification (Figure 4). In comparison to SpliceAI which was designed to predict a single canonical set of splice sites for each gene, a novel residual CNN‐based method, called Splam, uses a much shorter flanking context sequence to predict the splice sites (Chao et al., 2024). Its core is a designed deep grouped residual CNN model. Different from SpliceAI which relies on a window of 10,000 base pairs on each side of the splice site to achieve the highest accuracy, Splam focuses on a relatively limited window of 400 base pairs around the splice site and trains the donor and acceptor site pairs together, following the principle that the splicing mechanism recognizes both ends of each intron simultaneously. Particularly, Splam outperformed other models, including SpliceAI, especially in plant dataset, for example, using *Arabidopsis* intron and flanking regions sequences as training data. Transformers, with their self‐attention mechanisms, have shown promise in capturing long‐range dependencies in RNA sequences. SpliceTransformer (SpTransformer), which was a deep‐learning framework that predicts tissue‐specific RNA splicing alterations linked to human diseases based on genomic sequence and outperforms all previous methods on splicing prediction (You et al., 2024). Sptrasfromer is a large deep neural network model consisting of two main modules: an encoder module and a transformer module. The encoder module is consisting of multiple residual networks like ResNet structure, while encoder module uses the Sinkhorn Transformer, a variant of the transformer module allowing for handle longer sequences, for example, >8000 nt. Using similar strategy, Jónsson et al. (2024) proposed a more insightful methods, Spliceformer, which generates embeddings with residual neural networks and apply hard attention to select splice site candidates, enabling efficient training on long sequences, surpassing the leading tool, SpliceAI, in detecting splice sites.

Obviously, most of these CNN and transformer‐based DL models for alternative splicing prediction were performed only on human beings or some model plants. For important crops, it is still basically a blank. The delayed application of DL to plant alternative splicing, compared to animals, stems from several biological hurdles unique to plant systems. First, plant splice sites are notably weak and degenerate, with a higher frequency of non‐canonical junctions (e.g., GC–AG) and less conserved consensus sequences, particularly in GC‐ and U12‐type introns (Pucker & Brockington, 2018; Schuler, 2008). Second, the distinct compositional architecture—AT‐rich introns and GC‐rich exons—increases the risk of cryptic splice site recognition (Brown & Simpson, 1998). Third, plants exhibit highly stress‐responsive splicing regulation mediated by expanded families of splicing factors (e.g., SR proteins) with divergent RNA‐binding specificities (Y. Du et al., 2024). Fourth, the lack of large‐scale, long‐read validated isoform annotations in most crops leads to noisy training labels for supervised models (F. Xu et al., 2024). To adapt existing models (e.g., Splam, SpTransformer, and Spliceformer) for crop‐specific datasets, we propose several strategies. (1) Transfer learning: pre‐train on model plants and fine‐tune using high‐confidence splice sites from crop long‐read RNA‐Seq. (2) Feature enrichment: extend input encoding to include intronic AT content and proximity to U‐rich/UC‐rich enhancers. (3) Tailored negative sampling: use stricter distance thresholds (>20 nt from true splice sites) to avoid ambiguous labels due to abundant cryptic sites in plant introns. (4) Cross‐species validation: train on well‐annotated model plants, evaluate on less‐annotated crops, and apply active learning with a few manually curated junctions per target species. Performance should be benchmarked against long‐read datasets (e.g., PacBio/ONT). Additionally, DNA LLMs have recently achieved significant progress in plant genomics—for example, cross‐species splice site prediction—making LLM‐based approaches a promising future direction (Zhai, Gokaslan et al., 2025).

### 3.3 *cis*‐Regulatory elements prediction

**FIGURE 5:**
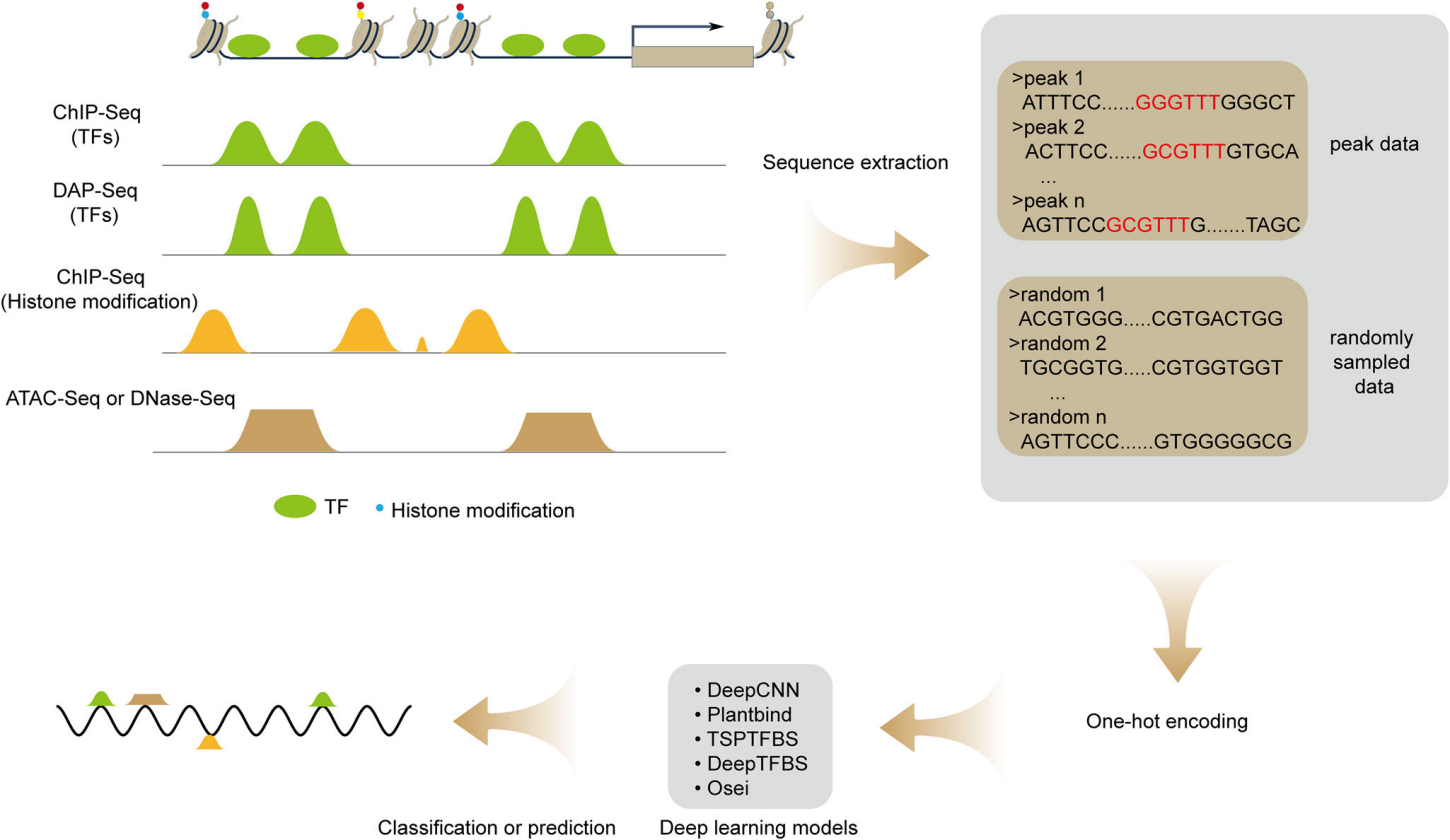
Schematic diagram of *cis*‐regulatory elements or epigenomics prediction using deep learning. The process begins with the acquisition of genome‐wide data for specific elements, such as transcription factor binding sites or histone modifications, through high‐throughput sequencing techniques like ChIP‐Seq. These experiments yield “peak” regions, which are used as positive samples representing functional genomic elements. For model training, an equivalent set of negative samples is generated, often by randomly sampling sequences from the nonpeak regions of the genome or by shuffling the genomic background. These DNA sequences (both positive and negative) are then converted into a numerical format, like one‐hot encoding, that the deep learning model can process. The resulting encoded data is fed into a deep learning model (e.g., a convolutional neural network [CNN]). The model learns to discern the complex sequence patterns and features associated with the regulatory elements. Once trained, the model can take a new, unlabeled DNA sequence as input, process it through the same one‐hot encoding and network architecture, and output a prediction score indicating the likelihood of the sequence containing the specific regulatory feature.

Although important DNA sequences or some motifs could be learned from feature importance analysis of DL‐based gene expression prediction in plants, it is not always a direct method for accurately identification of *cis*‐regulatory elements. Given a large scale of high‐throughput sequenced‐based TF‐binding data (e.g., ChIP‐Seq and CUT&Tag) available from model plants or crops, it has become feasible to use DL to decipher the regulatory codes from these valuable resources and to further predict novel *cis*‐regulatory elements such as TFBS, enhancers in other species (Shen et al., 2021) (Table 1). By learning the characteristics of transcription factor binding sequences, the model can identify the key sequence elements related to the binding of specific transcription factors (Figure 5). First example in plant TF‐binding prediction was performed by L. Liu, Zhang, et al. (2021) that employed the deep CNN (DeepCNN) to build 265 *Arabidopsis* TFBS prediction models based on available DNA affinity purification sequencing. They revealed that DeepCNN not only achieves greater successes on *Arabidopsis* TFBS predictions when compared with gkmSVM and multiple em for motif elicitation (MEME) but also has learned its known motif for most *Arabidopsis* TFs as well as cooperative TF motifs with protein–protein interaction evidences as its biological interpretability. Similar with this study, PlantBind, a method for integrated prediction and interpretation of TFBSs based on DNA sequences and DNA shape profiles was built on an attention‐based multi‐label DL framework. It not only simultaneously predicts the potential binding sites for from various plant TFs, but also identifies the motifs bound by transcription factors. Trans‐species prediction performance using four maize TFs demonstrated the suitability of this model for transfer learning (W. Yan et al., 2022). TSPTFBS 2.0 used DenseNet to perform TFBS prediction, and the authors combined three interpretability methods to identify the potential core motif within a TFBS, which will be a powerful tool for assisting plant scientists on providing candidate targets of genome editing (H. Cheng et al., 2023). DeepTFBS, another DL model of TF binding grammar for accurately predicting TFBSs within and across plant species. DeepTFBS can also utilize information from gene conservation and binding motifs, enabling efficient TFBS prediction in species where experimental data availability is limited (Zhai, Zhang et al., 2025). Therefore, by leveraging large‐scale sequencing data, DL models are effectively learning the grammar of transcriptional regulation in plants. This knowledge enables the reverse design of *cis*‐regulatory elements, offering a novel breeding strategy to control complex agricultural traits with unprecedented precision.

### 3.4 Epigenomic prediction

Epigenetic modifications play crucial roles in plant development, growth, and responses to environmental stimuli. With the rapid accumulation of high‐throughput epigenetic data in plants, DL has emerged as a powerful tool for predicting various epigenetic features, including DNA methylation, histone modifications, and chromatin accessibility (Table 1). An example of DNA methylation prediction based on DL is from a genomic DNA methylation map of 207 cotton accessions. A deep‐learning model, DeepFDML, was trained based on the sequences near DNA methylation sites to predict functional epigenetic modification sites. This research provides an important theoretical basis and technical support for the genetic improvement and variety selection of cotton (T. Zhao et al., 2024).  PlantDeepMeth, a novel DL model, was used to predict DNA methylation states in plants. Motif analysis of the model's predictions identified specific motifs associated with hypo‐ or hyper‐methylation states in *Brassica rapa* L. and *Arabidopsis* (Z. Guo et al., 2025). In addition to DNA methylation, a smart model for epigenetics in plants was built to predict six types of epigenomic modifications, including DNA 5‐methylcytosine and N6‐methyladenosine methylation, RNA N6‐methyladenosine methylation, and three types of histone modification in rice (*Oryza sativa* L.) genome (Y. Wang, Zhang, et al., 2021).

Histone modifications, such as H3K4me3 and H3K27me3, are essential for chromatin remodeling and gene expression regulation (Z. Wu et al., 2019). DL models have been specifically developed to predict these modifications based on DNA sequence and chromatin accessibility data (Figure 5). For instance, a deep‐learning model named Osei, was developed to analyze and predict multiple chromatin features in the rice genome, including TFBS, histone modification signals, and chromatin open regions (X. Zhou et al., 2024). The model can not only accurately classify regulatory sequences but also quantify the impact of genetic variations on regulatory activity changes and their effects on agronomic traits, providing new ideas for the future design of artificially synthesized regulatory sequences and the improvement of crop performance.

### 3.5 Genomic prediction or selection using population genetic data

Crop genomes carry the genetic information that determines various traits of plants, including yield, disease resistance, and stress tolerance. Understanding and predicting crop genomes are crucial for modern agriculture. With the development of high‐throughput sequencing technologies, vast amounts of genomic data have been generated, providing valuable data for genomic prediction. Traditional statistical methods like best‐linear unbiased prediction (BLUP) have been widely used in crop genome prediction before the application of deep‐learning methods (VanRaden, 2008; X. Wang et al., 2015). BLUP estimates the breeding values of individuals based on the genetic relationships among them. For example, it uses the kinship matrix calculated from single nucleotide polymorphism (SNP) data to predict the performance of offspring. However, traditional methods have limitations. They assume linear relationships between genomic features and traits, which may not hold true in many cases. Also, they often struggle to handle the high‐dimensionality of genomic data effectively.

**FIGURE 6:**
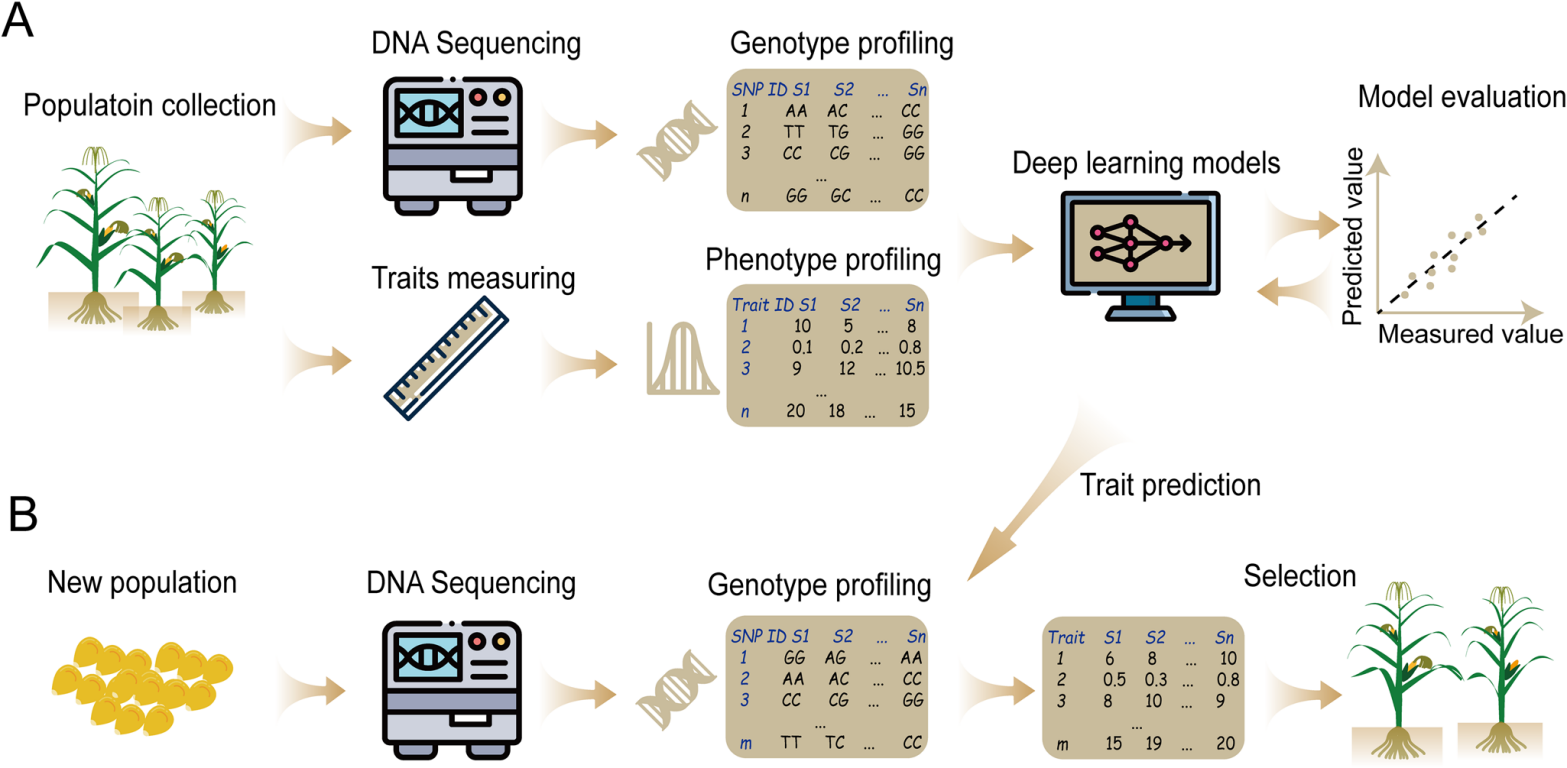
Schematic diagram of the genome‐wide prediction and selection pipeline using deep learning. The workflow is divided into two primary phases: (A) model training and (B) genomic prediction and selection. (A) The model training phase begins with the collection of a reference population (e.g., a natural population of maize), which undergoes high‐throughput DNA sequencing and rigorous phenotyping. The sequencing data is processed to call genotypes, such as single nucleotide polymorphisms (SNPs). Subsequently, these genotypic data are integrated with the phenotypic records. This integrated dataset is partitioned into training and validation sets to develop and tune deep learning models capable of capturing the nonlinear relationships between genetic markers and phenotypic traits. Model evaluation guides the selection of the optimal predictive algorithm. (B) In the genomic prediction phase, the best‐performing model is deployed to predict the breeding values of individuals in a new, untested population solely from their genotypic data, bypassing resource‐intensive phenotyping. Based on these predictions, elite candidates with the most promising trait values are prioritized and advanced for selective breeding in the next generation.

DL has the potential to revolutionize crop genome prediction, enabling more accurate and efficient breeding strategies. It can be used to predict agronomic traits such as yield, quality, and stress resistance in crop breeding. By integrating crop genotypic data (such as SNP markers), phenotypic data, and environmental data, a DL prediction model can be constructed (Figure 6; Table 1). For example, Khaki & Wang (2019) designed a deep neural network method to predict maize yield across 2247 locations from 2008 to 2016 using genotype and environmental data in the yield testing stage. Similarly, deep neural network genomic prediction (DNNGP), was developed and applied to a variety of omics data to predict phenotypes (K. Wang et al., 2023). DNNGP was tested on three plant species (wheat [*Triticum aestivum* L.], maize, and tomato) and compared against five established methods: genomic best‐linear unbiased prediction (GBLUP), light gradient boosting machine (LightGBM), support vector regression (SVR), deep genomic selection (DeepGS), and deep learning genome‐wide association study (DLGWAS). It demonstrated superior or competitive prediction accuracy, particularly excelling with large‐scale breeding data while maintaining strong performance on smaller datasets. Cropformer, another DL framework for predicting crop phenotypes and exploring downstream tasks. This framework combines CNNs with multiple self‐attention mechanisms to improve prediction accuracy (H. Wang et al., 2025). While TrG2P demonstrates the power of leveraging auxiliary phenotypic data to improve predictions for complex traits like yield, a critical frontier in breeding lies in accounting for the dynamic interplay between genotypes and their environments. The newly developed GEFormer directly addresses this gap by introducing a DL architecture that specifically models genotype‐by‐environment (G×E) interactions (Yao et al., 2025). Its core innovation lies in a dual‐stream fusion network: one stream employs a gated multilayer perceptron to capture both local and global dependencies among SNPs, while the other utilizes omni‐dimensional dynamic convolution and a linear attention mechanism to extract comprehensive daily features and temporal patterns from environmental data streams. Critically, these features are not merely concatenated. It is a gating mechanism that dynamically fuses the genomic and environmental representations, enabling the model to learn their complex, nonlinear interactions. This architecture moves beyond static genomic prediction by integrating the real‐world growth conditions that ultimately shape crop phenotypes, making it particularly powerful for predicting performance in untested environments. These advancements collectively signal a paradigm shift in crop breeding, from a largely experience‐dependent selection process to a data‐driven predictive science, ultimately accelerating the development of superior crop varieties.

## 4 APPLICATIONS OF DL IN PLANT PHENOMICS

### 4.1 Development of plant phenomics

**FIGURE 7:**
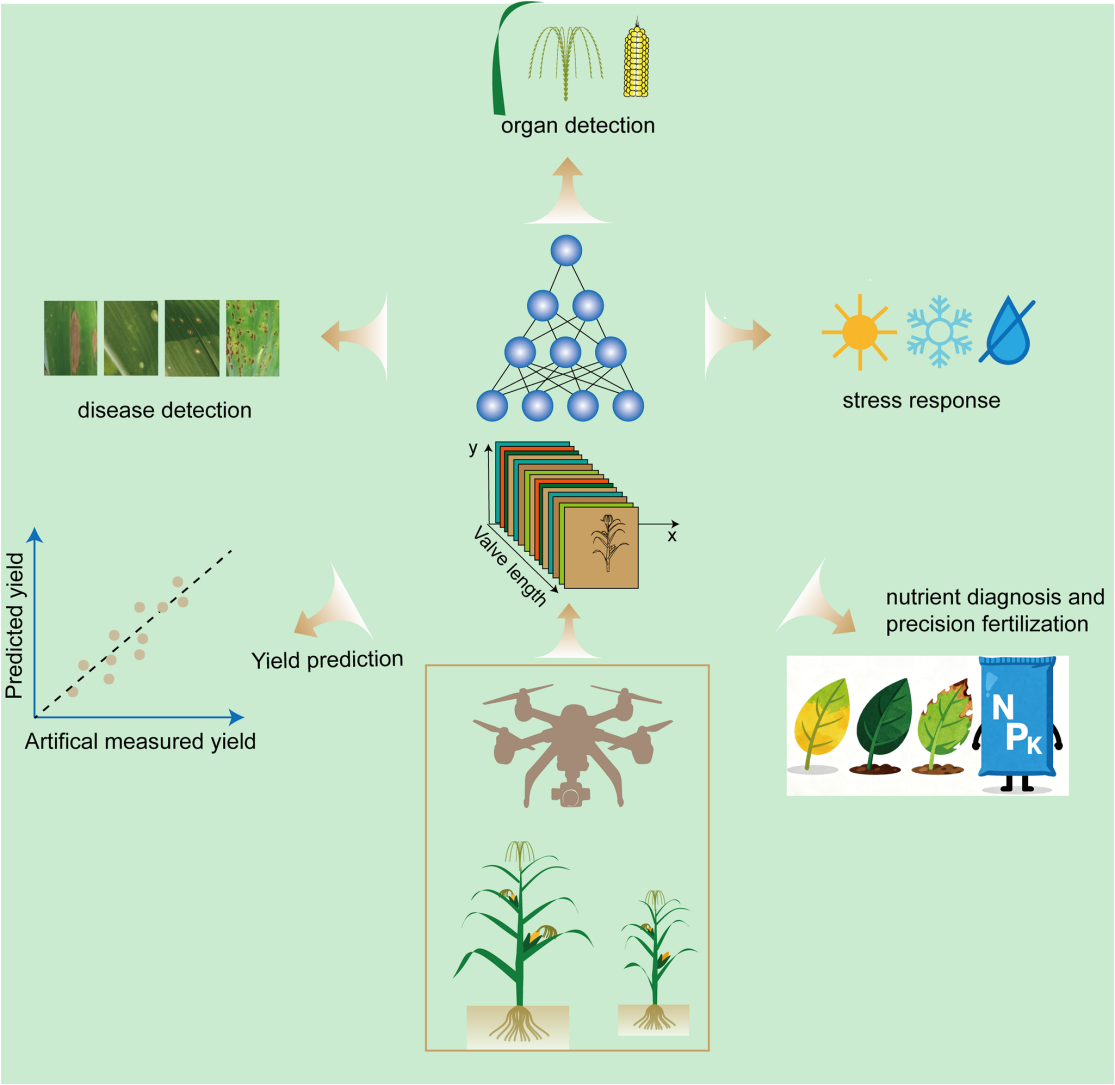
Application of crop canopy spectral analysis and inversion techniques. Unmanned aerial vehicles (UAVs) acquire multispectral or hyperspectral data across extensive field areas, which are subsequently processed by deep learning models to support crop phenomics research. Deep learning architectures—such as convolutional neural networks (CNNs) and Transformers—leverage these spatial‐spectral data cubes to extract latent features associated with crop physiological status, model complex nonlinear relationships between spectral signatures and phenotypic traits, and enable high‐throughput phenotyping at the plot or individual plant level. Key applications include mid‐season yield prediction based on spectral patterns, along with early identification of nutrient deficiencies, disease classification, and abiotic or biotic stress assessment.

Plant phenomics has emerged as a pivotal discipline that systematically studies the physiological and architectural traits of plants (phenotypes) throughout their growth cycle, in response to genetic makeup and environmental influences (C. Zhao et al., 2019). Traditionally, phenotyping relied heavily on manual measurements, which were often labor‐intensive, time‐consuming, and subjective, leading to a significant bottleneck in the genetic analysis and breeding process, famously known as the “phenotyping bottleneck” (Furbank & Tester, 2011). The advent of advanced intelligent devices and sensor technologies has revolutionized this field, driving the development of HTP platforms (Y. Cheng et al., 2025). The HTP platforms, encompassing ground‐based rovers, UAVs (or drones), and fully automated greenhouse systems, are equipped with a suite of sophisticated sensors, including high‐resolution RGB cameras, multi‐and hyperspectral sensors, light detection and ranging (LiDAR), thermal infrared cameras, and fluorescence imagers (Lankada et al., 2026). This technological synergy enables the rapid, nondestructive, and precise quantification of a wide array of phenotypic traits—from basic plant height and canopy coverage to complex physiological parameters such as photosynthetic efficiency, water stress status, and biomass accumulation—at an unprecedented scale and resolution. With these datasets, different DL methods can be applied to extract meaningful information from large quantities of image data, such as organ detection (James et al., 2023; B. Liu et al., 2024; Ullah et al., 2024), leaf area index estimation (Castro‐Valdecantos et al., 2022; S. Liu, Jin, et al., 2021; D. Zhao et al., 2025), plant height and biomass prediction, root architecture quantification (Teramoto & Uga, 2020; Weihs et al., 2024), disease detection (Dong et al., 2024; Toda & Okura, 2019), nutrient deficiency classification (Urfan et al., 2024; J. Wang, Chu, et al., 2024), and abiotic stress response (Walsh et al., 2024; Zagorscak et al., 2025; J. Zhou et al., 2021) (Figure 7; Table 1). Although DL in image‐based plant phenotyping has been reviewed in other publications (Murphy et al., 2024; Singh et al., 2018), here, we mainly focus on the applications of DL in two main aspects: crop yield prediction and disease detection.

### 4.2 Crop yield prediction based on phenomics

Crop yield must urgently be sustainably increased to accommodate a rising global population and anticipated climate change in the coming decades, in the face of plant stresses and limited resources. Traditional yield assessment methods have long relied on field sampling, including destructive approaches (e.g., manual harvesting of quadrats) and nondestructive measurements (e.g., yield component counting), which are labor‐intensive and limited in spatial representation. Recent years have witnessed transformative progress in crop yield prediction through remote sensing and image‐based approaches. Modern satellite platforms (Sentinel‐2 and Landsat 9) provide multispectral data at 10–30 m resolution, while UAVs enable centimeter‐scale imaging through RGB, multispectral (five to six bands), and hyperspectral (300+ bands) sensors (Trevisiol et al., 2024). Using these image data, researches could use different machine learning models to perform yield assessment (Table 1). For example, Tanaka et al. (2023) develops DL framework CNN that achieves real‐time rice yield estimation from ground‐based RGB images with over 70% accuracy, eliminating the need for complex sensors or manual measurements. Similar research was performed by H. Zhou, Huang, et al. (2025). In addition to rice, the yield estimation of wheat and soybean (*Glycine max* (L.) Merr.) could also be performed via different DL models using UAV captured image data (E. Cheng et al., 2022; Okada et al., 2024; L. Zhou, Han, et al., 2025). DL models based on CNN have also been used in other economic crops, such as orchard fruit (Apolo‐Apolo et al., 2020), tea (*Camellia sinensis* L.) (S. Wang, Yu, et al., 2024), and strawberry (*Fragaria × ananassa* Duch.) (C. Zheng et al., 2022). Particularly, transformer or transformer‐based DL models were confirmed to perform better than CNN‐based models in similar task (J. Du et al., 2025; Ge et al., 2025; F. Guo et al., 2024).

### 4.3 Disease detection of crops based on phenomics

DL and computer vision have become emerging tools for diseased plant phenotyping. For example, Toda and Okura (2019) used CNN models to distinguish the different plant diseases and healthy using the image data. Moreover, the models could capture the colors and textures of lesions specific to respective diseases upon diagnosis via a variety of neuron‐wise and layer‐wise visualization methods. Similarly, a plant phenomics analysis of disease (PlantPAD) platform (http://plantpad.samlab.cn) with large‐scale information on disease was developed, which covers 421,314 images, 63 crops, and 310 diseases, and integrates more than 20 DL models for plant disease diagnosis (Dong et al., 2024). In addition to canonical CNN methods, Batool et al. (2025) built a novel DL model, depthwise separable convolution with spatial attention (LWDSC‐SA), which could enhance feature extraction while maintaining computational efficiency in plant disease classification. While DL and computer vision are emerging in diseased plant phenotyping with prior work mainly on image‐level disease classification, L. Zhou et al. (2023) analyzed pixel‐level phenotypic features by collecting a diseased leaf dataset using apple leaf samples for training and grape/strawberry samples for extra testing.

## 5 BRIDGING GENOMICS AND PHENOMICS: AN INTEGRATIVE FRAMEWORK AND ROADMAP FOR CROP RESEARCH

**FIGURE 8:**
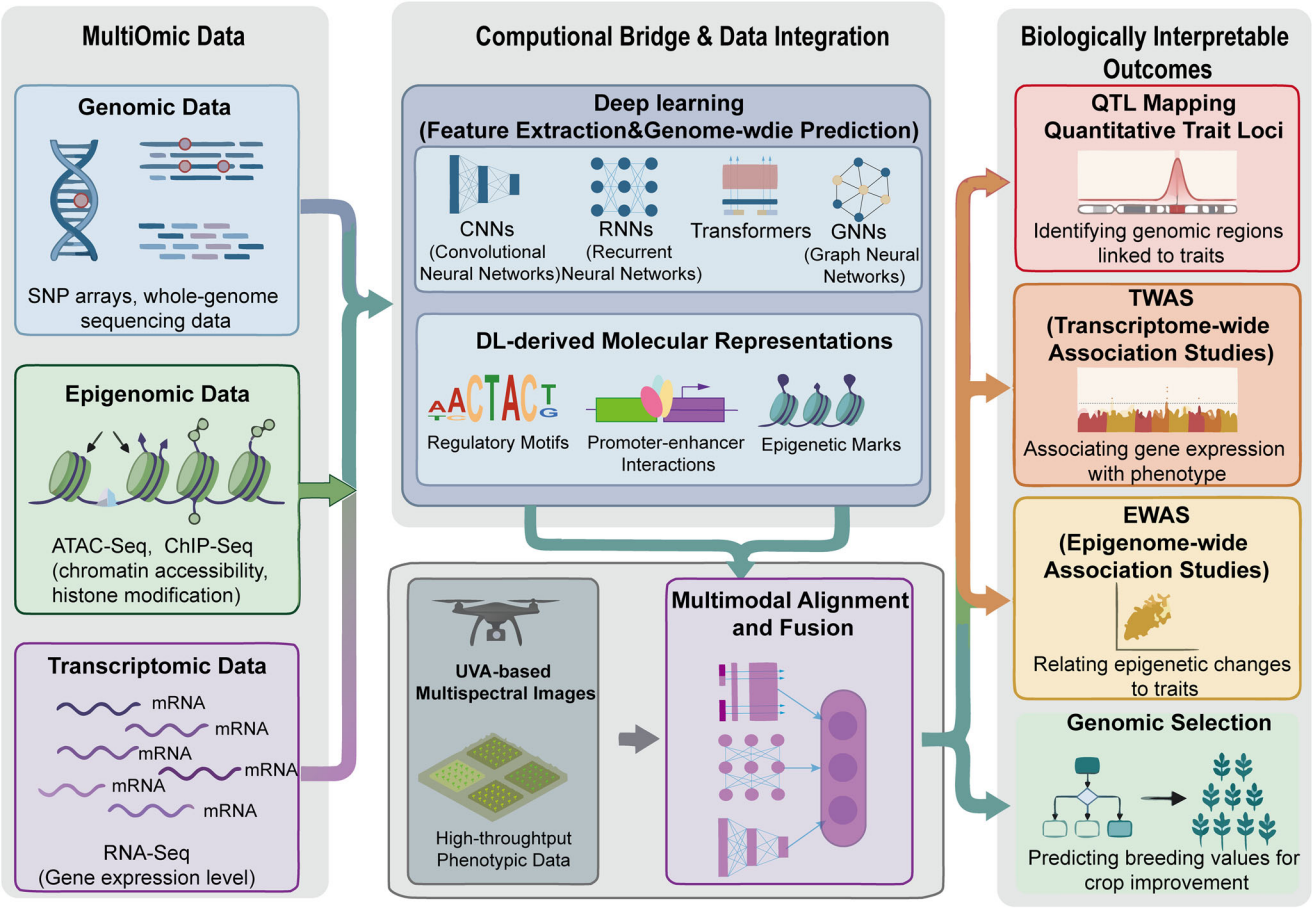
Bridging multi‐omics and phenomics with deep learning and multimodal fusion framework. At different omics levels (such as genomics, epigenomics, and transcriptomics), researchers can leverage large amounts of relevant data to construct deep learning models, thereby extracting features and integrating multiple modalities of data sets. Subsequently, the phenotypic data set and the aligned multimodal data set are subjected to another round of deep learning analysis, which fully exploits multilevel biological information. This lays a solid foundation for elucidating the genetic and biological mechanisms of crops, identifying key loci controlling important agronomic traits, and conducting genomic selection in crop breeding.

Typically, the approach to link phenotype with genotypes in crops involves using high‐throughput phenomics values as the target variable and genotypic data (e.g. SNPs and indels) as predictor variables in genome‐wide association studies (GWAS) or building DL‐based genomic selection models (J. Chen et al., 2024; Gan et al., 2025; Jiang et al., 2021; J. Wang, Li, et al., 2021; H. Zheng et al., 2024). However, these studies often overlook information from other molecular levels. Epigenomic data (e.g., ATAC‑seq and ChIP‑seq for chromatin accessibility and histone modifications) and transcriptomic data (e.g., RNA‑seq for gene expression levels) each provide distinct molecular layers that influence crop phenotypes. DL models, particularly CNNs, RNNs, transformers, and GNN, can extract hierarchical features from these multi‑omics data, such as identifying regulatory sequence motifs from DNA, predicting gene expression from promoter‑enhancer interactions, or capturing epigenetic marks that correlate with stress responses. These DL‑derived molecular representations can be integrated with high‑throughput phenotypic data (e.g., UAV‑based multispectral images and ground‑robot‑derived trait vectors) to create a unified framework that links genotype to phenotype. Such a multi‑omics‑to‑prediction data flow, illustrated in Figure 8, positions DL as the computational bridge that transforms raw molecular data (genomic, epigenomic, and transcriptomic) and sensor‑based phenomics data into biologically interpretable outcomes—enabling quantitative trait locus (QTL) mapping, transcriptome‑wide association studies, epigenome‑wide association studies, and genomic selection. By aligning these diverse data modalities within a shared latent space, researchers can uncover causal regulatory networks and accelerate breeding for complex agronomic traits.

## 6 CHALLENGES

### 6.1 Data requirements and quality

DL models usually require a large amount of high‐quality data for training. However, the acquisition of plant or crop genome data is often limited by experimental techniques, sample numbers, and data quality control. According to our knowledge, most of present DL models are mainly built in model plants, such as *Arabidopsis*, rice, and maize, which was mainly due to a large scale of omics data available in these plants (Scossa et al., 2021). While for non‐model plants or crops, the genome data are scarce, and the existing data may not many enough for training models, which will affect the performance and reliability of DL models. To address the data scarcity in orphan crops, several practical strategies can be adopted: constructing pan‑genomes to capture full genetic diversity, transferring functional annotations from phylogenetically related model crops, optimizing training population design under limited phenotyping capacity, and employing low‑coverage sequencing for SNP discovery. Among these, leveraging pretrained DNA foundation models for cross‑species transfer learning offers a particularly efficient route to compensate for limited genomic resources. Recent plant DNA foundation models enable effective transfer learning for orphan crops. AgroNT (pretrained on 48 edible plants) uses parameter‑efficient fine‑tuning and has been successfully applied to cassava (Mendoza‐Revilla et al., 2024). DNABERT‑2 captures cross‑species conserved features via self‑attention and outperforms traditional SNP‑based methods for phenotype prediction (Ji et al., 2021). Most notably, PlantCaduceus—fine‑tuned on only a small set of *Arabidopsis* data—transferred successfully to maize (despite 160 million years of divergence), improving splice donor prediction by 1.45‑fold (Zhai, Gokaslan et al., 2025). These open‑source models make cross‑species transfer feasible even for breeding programs with limited computational infrastructure.

The future of AI in plant biology hinges on data excellence. High‐quality, FAIR (findable, accessible, interoperable, and reusable)‑compliant data with rigorous experimental design and MIAPPE (minimum information about plant phenotyping experiments)‑standardized metadata are essential for training generalizable DL models (Wilkinson et al., 2016). We therefore recommend depositing plant phenotyping and multi‐omics data in repositories that actively support MIAPPE and FAIR compliance. These include the European Nucleotide Archive, NCBI BioSample, GigaDB, e!DAL (electronic Data Archive Library), and so on. When publishing, authors should provide a MIAPPE‑compliant metadata file, cite the dataset with a persistent DOI, and link it to corresponding genomic or phenotypic publications. Such practices will greatly enhance data reusability, facilitate cross‑study meta‑analyses, and accelerate the development of robust DL models in crop science.

### 6.2 Model interpretability

The increasing complexity and opacity of machine learning models make interpretability essential and deciphering model logic is now both a key research direction and a practical requirement in applied AI. In crop research, understanding how a model arrives at a specific prediction result is crucial for revealing biological mechanisms.

Feature importance analysis stands as a cornerstone technique within this field, aiming to quantify the contribution of individual input features or units toward a model's output. Interpretability or feature importance analysis methods can be classified into different categories, including scope, model‐agnosticism, and technical approach (Table 2). Generally, they are broadly categorized into intrinsic (designing inherently interpretable models) and post hoc (explaining existing black‐box models), with post hoc techniques dominating DL interpretability research (Lipton, 2018). The following subsections focus on post hoc methods categorized by their technical approach, as summarized in Table 2.

**TABLE 2:** A summary of feature importance analysis methods. in deep learning models

Category	Core idea	Representative methods	Application	Publications
Gradient‐based	Use gradients to measure importance	Saliency maps	CNN for DNA sequence classification, DenseNet for phenotyping, CNN for disease image classification	(Gao et al., 2018; Nagasubramanian et al., 2020; Shoaib et al., 2023)
		Grad‐CAM	CNN for disease image classification	(Ahmmed et al., 2026; Ennadifi et al., 2020; Murugesan et al., 2025)
		Integrated gradients	DenseNet for phenotyping, ResNet + BiLSTM for TFBS prediction	(Nagasubramanian et al., 2020; Zhai, Zhang et al., 2025)
Perturbation‐based	Modify input and observe output	LIME (local interpretable model‐agnostic explanation)	CNN for disease image classification	(Ahmmed et al., 2026)
		SHAP	Genomic prediction	(He et al., 2025; J. Li et al., 2026; Ruggeri & Vidács, 2025)
Surrogate Models	Approximate with a simple model	Global surrogates	DNN for sequence classification	(Seitz et al., 2024)
		LIME	CNN for disease image classification	(Ahmmed et al., 2026)
Activation‐based	Analyze internal neuron activations	Activation maximization	Optimization of DNA and protein sequences	(Linder & Seelig, 2021)
Propagation‐based	Backpropagate relevance scores layer‐wise	Layer‐wise relevance propagation (LRP)	DenseNet for phenotyping	(Nagasubramanian et al., 2020)
		DeepLIFT	ResNet for DNA sequence classification	(H. Cheng et al., 2023; Peleke et al., 2024)
Attention‐based	Leverage attention weights to interpret model decisions	Self‑attention weights	Genomic Prediction	(H. Wang et al., 2025)

Abbreviations: BiLSTM, bidirectional long short‐term memory network; CNN, convolutional neural network; ResNet, residual convolutional network; SHAP, Shapley additive explanations; TFBS, transcription factor binding sites.

Gradient‐based methods leverage the model's gradients to estimate feature importance. Saliency maps visualize input sensitivity by computing gradients of the output class score with respect to the input (Simonyan et al., 2013), and has been widely used to explain or interpret DL modes like CNN for DNA sequence or image classifications in crop research (Gao et al., 2018; Nagasubramanian et al., 2020; Shoaib et al., 2023). Grad‑CAM (gradient‑based generalization of class activation mapping) generates coarse localization heatmaps by combining convolutional feature maps and gradients, and is widely used for image‐based crop disease classification (Ahmmed et al., 2026; Ennadifi et al., 2020; Murugesan et al., 2025). Integrated gradients (IG) address saturation and sensitivity issues by integrating gradients along a path from a baseline input to the actual input and were used in both genomic DNA model interpretation and phenomics study (Nagasubramanian et al., 2020; Sundararajan et al., 2017; Zhai, Zhang et al., 2025). Perturbation‐based methods assess importance by observing output changes upon modifying inputs. LIME (Local Interpretable Model‐agnostic Explanations) approximates the local decision boundary of the complex model by learning a simple, interpretable surrogate model (e.g., linear regression) on perturbed samples around a specific prediction, providing local feature weights (Ribeiro et al., 2016). This method was also used to explain the DL models for crop disease classification (Ahmmed et al., 2026). Shapley additive explanations (SHAP) leverage cooperative game theory (Shapley values) to fairly allocate feature contributions and provides a unified additive framework that generalizes LIME, DeepLIFT, and other local explanation methods (Lundberg & Lee, 2017). SHAP has been widely used to identify important SNPs controlling agronomy traits in crop genomic prediction models (He et al., 2025; R. Li, Zhang, et al., 2025; Ruggeri & Vidács, 2025). Surrogate model methods globally approximate the complex model with a simpler interpretable model (e.g., decision tree and linear model) trained on the original model's predictions, offering global insights but potentially sacrificing fidelity (Diaw et al., 2024). Some researchers have used this method to explain DL modes for both genomic DNA and image classifications (Ahmmed et al., 2026; Seitz et al., 2024). Activation‑based methods aim to understand what a neural network has learned by analyzing the internal activations of its neurons. Instead of explaining a single prediction, these methods seek to generate synthetic inputs that maximize the activation of a specific neuron, channel, or layer. The resulting synthetic pattern reveals the type of input features that the neuron responds to most strongly. Although seldom used this method, it could be used in optimize DNA or protein sequences in genomics (Linder & Seelig, 2021). Propagation‑based methods backpropagate relevance scores from the output to the input layer without relying on gradients. Layer‑wise relevance propagation (LRP) decomposes the prediction output backward through the network layers using specific propagation rules to assign relevance scores to input features, applicable to various architectures including CNNs and LSTMs (Binder et al., 2016; Nagasubramanian et al., 2020). DeepLIFT, another propogation‐based methods, which compares neuron activations to a “reference” activation, propagating differences backward to assign importance scores (Shrikumar et al., 2017). This method has been widely used in DNA sequence classification models (H. Cheng et al., 2023; Peleke et al., 2024). Since the development of transformer‐based DL models, attention‐based model explanation methods have gradually gained trust, and it has been used to explain transformed‐based models in crop study (H. Wang et al., 2025). Software toolkits have become essential for practical application. Major software or libraries include SHAP, offering efficient implementations for various models including DL (Lundberg & Lee, S.‐I., 2017); Captum (Facebook AI), providing PyTorch‐native implementations of IG, DeepLIFT, LRP, Grad‐CAM, and many others (Kokhlikyan et al., 2020); tf‐explain (https://github.com/sicara/tf‐explain), offering similar methods for TensorFlow/Keras; LRP Toolbox, specifically optimized for LRP (Lapuschkin et al., 2016); iNNvestigate, offering a unified API for multiple methods in Keras (Alber et al., 2018); ELI5 (https://github.com/eli5‐org/eli5) supporting text/image explanations and model inspection; Alibi focusing on high‐quality, scalable implementations including Anchor explanations and Counterfactuals (https://github.com/SeldonIO/alibi); InterpretML (Microsoft) offering glass‐box models and explainers like SHAP and LIME (https://github.com/interpretml/interpret); and DeepExplain, providing a unified framework for state‐of‐the‐art gradient and perturbation‐based attribution methods (https://github.com/marcoancona/DeepExplain).

Despite the success of DL models in linking genotype to phenotype in crop studies, model opaqueness remains a major barrier to adoption in plant science. Evaluating explainable AI methods requires assessing whether explanations are faithful (truly reflecting model reasoning) and robust (stable under small input perturbations). However, the lack of standardized quantitative metrics means that validation often defaults to subjective judgment or task‑specific checks such as pathway enrichment. Without rigorous and systematic benchmarking, it remains difficult to trust that highlighted genomic regions truly guide biological discovery rather than misleading functional validation efforts. For this issue, we propose a simple three‑step framework. First, use simulated data where we already know which genes or features actually control a trait (e.g., we deliberately set 10 key genes to affect yield) and then check whether the model's top‑ranked features correctly pick out those known genes, for instance, by counting how many of the true causal genes appear among the top‑10 model explanations. Second, for real experimental data, ask whether the features the model considers most important match existing biological knowledge: Do they fall inside known QTL regions? Are they expressed in the right tissue (such as roots, leaves, or grains) or at the right growth stage? Do they belong to well‑known metabolic pathways or regulatory networks? Third, and most convincingly, physically perturb the top‑ranked features (e.g., knock out a candidate gene, apply a chemical inhibitor, or use natural genetic variants) and see whether the model's prediction or the actual plant phenotype changes as expected. As a simple “biological truth” checklist, we recommend that every study reporting model interpretability answer four basic questions: are the top features located close to known QTLs (say within 50,000 base pairs)? Are they expressed in the relevant tissue or developmental stage? Are they more likely to share a biological pathway than random genes? And how do the model's explanations compare to those from a very simple baseline, such as a linear regression or a single‑marker association test? Using this tiered framework and checklist will help plant scientists move beyond black‑box curiosity and gain genuine biological confidence in their model interpretations.

### 6.3 Computational resources and efficiency

The substantial computational demands inherent in developing and deploying DL models represent a significant barrier to their broader adoption in plant genomics research. The computational burden is further amplified when dealing with polyploid genomes common in many crops (e.g., wheat and potato), which may potentially require more complex model representations and larger input sizes, or when employing computationally intensive interpretability methods (e.g., permutation‐based feature importance, SHAP, and complex saliency map generation) post‐training to understand model predictions on genomic features. In addition, although DL has achieved remarkable accuracy in plant phenotyping and genomic prediction, many state‑of‑the‑art models are too computationally heavy for on‑device deployment on drones, handheld sensors, or embedded systems, motivating the use of lightweight neural networks and model compression techniques. Lightweight architectures such as MobileNet, EfficientNet‑Lite, and ShuffleNet use depth‐wise separable convolutions or channel shuffling to drastically reduce parameter counts while maintaining competitive accuracy (Howard et al., 2017; C. Wang, Chiu, et al., 2020; X. Zhang et al., 2017). For example, MobileNetV3 achieves ImageNet accuracy comparable to ResNet‑50 with only ∼5% of the multiply‑accumulate operations, and these models have been successfully applied to on‑device plant disease detection and UAV‑based stress phenotyping. Model compression techniques include pruning (removing redundant weights or channels), quantization (reducing numerical precision, for example, from FP32 to INT8, which accelerates inference by 2–4x on edge hardware with minimal accuracy loss), and knowledge distillation (training a compact student model to mimic a larger teacher model). For practical deployment, breeders and plant scientists can use open‑source toolkits such as TensorFlow Lite, PyTorch Mobile, and ONNX Runtime. A typical UAV‑compatible platform like NVIDIA Jetson Nano or Raspberry Pi 4 can run a quantized MobileNet at 10–30 FPS, sufficient for real‑time field applications, thus lowering hardware barriers and enabling on‑device intelligence without relying on cloud connectivity (Hakani & Rawat, 2024).

## 7 FUTURE PERSPECTIVES

Despite the many challenges faced by DL in crop research, with the continuous development of technology, its future prospects remain very broad. The effective application of DL in crop genomics and phenomics hinges critically on addressing three interconnected pillars: (1) matching methodological approaches to specific problem types and data structures, (2) selecting or designing appropriate models for distinct datasets and tasks, and (3) the strategic curation of high‐quality, multimodal datasets. Future advancements will require deliberate progress in each area to unlock the full potential of DL for accelerating crop improvement.

### 7.1 Methodological suitability: Aligning DL architectures with problem types

For genomic sequence analysis, CNNs, particularly 1D‐CNNs, remain efficient for identifying local patterns in sequence data and have to some extent become a standard for analysis of biological sequences (Z. Zhang et al., 2021). Transformers show significant promise for capturing long‐range dependencies inherent in genome sequences due to their attention mechanism, which captures relationships across entire sequences, independent of nucleotide proximity (Avsec et al., 2021). However, it is important to note that the discussions and advancements in these sophisticated models have predominantly centered on animal and human genomic studies. Research applying these approaches to plant and crop genomes remains relatively scarce, despite their immense agricultural importance and unique architectural features, such as larger genome sizes and higher ploidy. Hybrid CNN‐Transformer architectures or genomic‐optimized transformer variants are anticipated (Yao et al., 2023). For functional genomics (e.g., promoters, enhancers, and ncRNA), while RNNs and LSTM networks capture sequential dependencies, attention‐based models (e.g. transformers) are increasingly favored for modeling long‐range regulatory interactions (Xiao et al., 2025). GNNs offer advantages for integrating known regulatory networks (e.g., gene‐transcription factor interactions) (Wei et al., 2024). For GWAS and genomic selection, traditional linear models, such as BLUP and GBLUP, have long served as the foundational framework (X. Wang et al., 2018). However, the expanding size of sequenced populations and the concomitant increase in data density pose a significant challenge for traditional analytical methods. DL models excel at modeling complex, nonlinear gene–gene and G×E interactions, especially with high‐dimensional genomic marker data (Alemu et al., 2024). In fact, different hybrid DL models have been applied to genome selection in multiple crops (Sandhu et al., 2022). Notably, R. Li, Zhang, et al., 2025 evaluated several hybrid DL models (CNN‐LSTM, CNN‐ResNet, LSTM‐ResNet, and CNN‐ResNet‐LSTM) for crop genomic selection and reported that the LSTM‐ResNet model yielded superior performance.

In the field of plant phenomics, CNNs, including ResNet and EfficientNet, serve as standard backbones in image analysis, such as organ detection, counting, size, color, and disease (Y. Jiang & Li, 2020; Y. Li et al., 2022; Luo et al., 2023; Warman et al., 2021). Instance segmentation models (Mask R‐CNN and YOLO variants) enable precise plant organ extraction (Sapkota et al., 2024). Temporal models (3D CNNs, RNNs/LSTMs, and transformers) are essential for growth dynamics and behavioral analysis (e.g., leaf movement) (Han & Wang, 2025). Deployment on edge devices demands lightweight models to overcome the high computational complexity and poor robustness in image‐based phenomics (P. Zhang et al., 2023). For point cloud/3D reconstruction, the point cloud neural networks (e.g., PointNet++ and PointTransformer) and 3D CNNs directly process LiDAR or Structure‐from‐Motion data (Maskeliūnas et al., 2025; Sorokin et al., 2024).

### 7.2 Model selection and optimization: Tailoring for agricultural data

The application of large‐scale foundation models is emerging as a transformative strategy for agricultural data science. Effective model selection and optimization in this domain thus necessitate specialized approaches, including domain adaptation via transfer learning, biologically informed architecture design, and a focus on computational efficiency. Here, domain‐specific pre‐training with foundation models supersedes generic approaches. For instance, genomic foundation models (e.g., DNABERT, nucleotide transformer, and AgroNT), pretrained on vast public datasets, provide powerful priors for efficient transfer to crop‐specific tasks like variant effect prediction, significantly reducing downstream data requirements (Consens et al., 2025; Ji et al., 2021; Zhai, Gokaslan et al., 2025). Similarly, agricultural vision models pretrained on diverse domain‐specific imagery (spanning crops, growth stages, lighting conditions, and backgrounds) outperform generic models when handling complex agricultural scenes with scale variations (Dong et al., 2023; Nahian et al., 2025; Zhu et al., 2025). Bridging domain gaps between controlled (lab/greenhouse) and complex field environments requires unsupervised/self‐supervised domain adaptation techniques—such as adversarial training and self‐training using unlabeled field data—coupled with few‐shot learning for rapid adaptation to new crops or traits. Incorporating biological priors—including gene pathways, metabolic networks, and spatiotemporal developmental rules—into model architectures via graph neural GNNs or loss functions (e.g., structural consistency constraints) enhances performance, generalization, and interpretability. Computational efficiency is critical for field deployment: model compression techniques like knowledge distillation, pruning, and quantization are imperative to run complex models on edge devices (drones, robots, and handhelds), while privacy‐preserving methods like federated learning enable cross‐institutional collaboration (e.g., among breeding companies) without sharing sensitive raw data.

### 7.3 Dataset curation: Synergizing quality, scale, and diversity

Strategic dataset curation for crop genomics and phenomics demands synergistic integration of quality, scale, and diversity, beginning with high‐quality annotation underpinned by adopting international standards (e.g., MIAPPE and Crop Ontology) for trait definition, measurement protocols, and genomic data annotation consistency (Papoutsoglou et al., 2020; Shrestha et al., 2010). Expert‐guided annotation, when integrated with crowdsourcing or semi‐supervised learning, effectively scales complex labeling tasks, while active learning enhances resource efficiency by intelligently selecting the most informative samples. Generative models, such as GANs and diffusion models, facilitate biologically realistic data augmentation—simulating variations in lighting, viewpoint, stress intensity, and genetic traits—to mitigate data scarcity for rare phenotypes or extreme environmental conditions. Addressing scale and diversity demands the development of integrated multimodal datasets that link multi‐omics data (genomics, transcriptomics, epigenomics, proteomics, and metabolomics) with multi‐scale phenomics (from cellular to population levels) to decode genotype–phenotype relationships (Zou et al., 2025). Environmental representativeness necessitates data collected across diverse geographies, seasons, years, and management practices to reliably model G×E interactions (Washburn et al., 2021). Similarly, genetic diversity must be captured through the inclusion of landraces, wild relatives, and structured populations (e.g., nested association mapping [NAM] and multiparent advanced generation intercross [MAGIC]) to ensure broad variability for generalizable models (Lazaridi et al., 2024). The implementation of open science depends on several key components: large, well‐annotated public repositories equipped with data privacy and IP protections; rich, ontology‐standardized metadata that is essential for experimental reproducibility; and scalable cloud‐based platforms capable of storing, processing, and analyzing multimodal agricultural biological data like Smart Breeding Platform (H. Li, Li, et al., 2024; H. Wu et al., 2025).

With the further integration of multi‐omics data (such as genomics, transcriptomics, proteomics, and metabolomics), DL models will be able to more comprehensively mine the information in plant genomes and deeply analyze the molecular mechanisms of plant life processes (Ballard et al., 2024; Y. Li, Wang, et al., 2025). However, most current models still neglect tissue‑specific and temporal dynamics during plant development. To overcome this limitation, we advocate integrating time‑series transcriptomics (e.g., RNA‑Seq across growth stages) with daily UAV‑based phenotyping (hyperspectral, thermal, or RGB). Such multimodal data can be fused using attention‑based recurrent networks (e.g., LSTM or transformers with cross‑modal attention) that align molecular trajectories with canopy‑level changes. For tissue‑specificity, collecting organ‑resolved transcriptomes (root, leaf, and stem) paired with corresponding organ‑level phenotypes allows multitask learning to predict stage‑specific traits. Practically, breeders could sample UAV imagery every 2–3 days and matching tissue samples for RNA‑seq, then train time‑aware models to predict yield or stress tolerance while interpreting attention weights to identify critical developmental windows and candidate genes.

The future integration of DL with emerging biotechnologies—particularly single‑cell sequencing and gene editing—offers a powerful pathway toward decoding the regulatory complexity of plant genomes at higher resolution. Single‑cell ATAC‑Seq (scATAC‑Seq), enabled by platforms such as scPlantReg, allows the identification of cell‑type‑specific accessible chromatin regions and inference of chromatin interactions from co‑accessibility patterns (H. Yan et al., 2026), while DL frameworks like Hi‑Compass can predict cell‑type‑specific 3D genome organization using accessibility data alone (Sun et al., 2026). Notably, polymer‑physics‑informed DL has been applied to maize, rice, and soybean to correct long‑range chromatin interaction predictions from single‑cell data, reducing false‑positive rates by up to 95% (Schlegel et al., 2025). To unlock the underexplored role of distal regulatory elements in crops, we envision combining scATAC‑Seq and scRNA‑Seq from the same tissues, training sequence‑to‑function DL models to predict cell‑type‑specific enhancer activity, and then using these predictions to nominate candidate elements for CRISPR (clustered regularly interspaced short palindromic repeats)‑based functional validation. DL‑assisted guide RNA design tools are now emerging for plants, enabling precise targeting of predicted regulatory sequences (Narra et al., 2025). This integrated pipeline, which combines single‑cell profiling and DL‑driven prediction of regulatory elements and 3D contacts followed by CRISPR validation, will transform how we study and engineer crop gene regulation.

## 8 CONCLUSIONS

DL has demonstrated transformative potential in bridging crop genomics and phenomics, enabling unprecedented capabilities in decoding genetic regulation and interpreting complex phenotypic traits, thereby driving the modernization of crop breeding toward intelligent and data‐driven paradigms. By leveraging models like CNNs, transformers, and GANs, researchers can predict gene expression, alternative splicing, *cis*‐regulatory elements, and epigenetic modifications with high accuracy, accelerating the discovery of genotype–phenotype relationships and supporting genomic selection in smart breeding programs. In phenomics, DL‐driven image analysis facilitates high‐throughput trait quantification, yield prediction, and disease diagnosis, enhancing precision and efficiency in breeding decisions. The integration of multimodal data and biologically informed modeling frameworks is increasingly forming the core of intelligent breeding systems, enabling more predictive and targeted crop improvement. Despite challenges—including data scarcity, model interpretability, and computational demands—ongoing innovations in multimodal data integration, biologically informed architectures, and efficient algorithms promise robust solutions tailored to real‐world breeding scenarios. Future advancements will depend on curated datasets, domain‐adapted models, and ethical AI frameworks to ensure scalability and adoption in global breeding efforts. Ultimately, DL‐powered tools are poised to revolutionize crop improvement, paving the way for smart breeding strategies that combine genomic selection with high‐resolution phenomic insights to achieve sustainable and climate‐resilient agriculture.

## AUTHOR CONTRIBUTIONS

**Zefeng Wu**: Conceptualization; formal analysis; funding acquisition; methodology; project administration; writing—original draft. **Yali Sun**: Data curation; funding acquisition; methodology; writing—original draft. **Qian Luo**: Data curation; formal analysis; visualization. **Jiaping Wei**: Investigation; resources. **Junmei Cui**: Data curation; formal analysis. **Yan Fang**: Formal analysis; investigation. **Yining Niu**: Investigation; validation. **Zhaohong Li**: Investigation; resources; validation. **Xiaolin Wang**: Data curation; visualization. **Zigang Liu**: Conceptualization; project administration.

## CONFLICT OF INTEREST STATEMENT

The authors declare no conflicts of interest.

## Data Availability

No data were used for the research described in the article.
